# Functional genomics of OCTN2 variants informs protein-specific variant effect predictor for Carnitine Transporter Deficiency

**DOI:** 10.1073/pnas.2210247119

**Published:** 2022-11-07

**Authors:** Megan L. Koleske, Gregory McInnes, Julia E. H. Brown, Neil Thomas, Keino Hutchinson, Marcus Y. Chin, Antoine Koehl, Michelle R. Arkin, Avner Schlessinger, Renata C. Gallagher, Yun S. Song, Russ B. Altman, Kathleen M. Giacomini

**Affiliations:** ^a^Department of Bioengineering and Therapeutic Sciences, University of California, San Francisco, CA 94143;; ^b^Biomedical Informatics Training Program, Stanford University, Stanford, CA 94305;; ^c^Empirico Inc., San Diego, CA 92122;; ^d^Program in Bioethics, University of California, San Francisco, CA 94143;; ^e^Institute for Health & Aging, University of California, San Francisco, CA 94143;; ^f^Computer Science Division, University of California, Berkeley, CA 94720;; ^g^Department of Pharmacological Sciences, Icahn School of Medicine at Mt. Sinai, New York, NY 10029;; ^h^Small Molecule Discovery Center, Department of Pharmaceutical Chemistry, University of California, San Francisco, CA 94143;; ^i^Department of Statistics, University of California, Berkeley, CA 94720;; ^j^Institute for Human Genetics, University of California, San Francisco, CA 94143;; ^k^Department of Pediatrics, University of California, San Francisco, CA 94143;; ^l^Department of Bioengineering, Stanford University, Stanford, CA 94305;; ^m^Department of Genetics, Stanford University, Stanford, CA 94305

**Keywords:** transporter, variant interpretation, machine learning, rare disease

## Abstract

Genetic variants in *SLC22A5*, encoding the membrane carnitine transporter OCTN2, cause the rare metabolic disorder Carnitine Transporter Deficiency (CTD). CTD is potentially lethal but actionable if detected early, with confirmatory diagnosis involving sequencing of *SLC22A5*. Interpretation of missense variants of uncertain significance (VUSs) is a major challenge. In this study, we sought to characterize the largest set to date (*n* = 150) of OCTN2 variants identified in diverse ancestral populations, with the goals of furthering our understanding of the mechanisms leading to OCTN2 loss-of-function (LOF) and creating a protein-specific variant effect prediction model for OCTN2 function. Uptake assays with ^14^C-carnitine revealed that 105 variants (70%) significantly reduced transport of carnitine compared to wild-type OCTN2, and 37 variants (25%) severely reduced function to less than 20%. All ancestral populations harbored LOF variants; 62% of green fluorescent protein (GFP)–tagged variants impaired OCTN2 localization to the plasma membrane of human embryonic kidney (HEK293T) cells, and subcellular localization significantly associated with function, revealing a major LOF mechanism of interest for CTD. With these data, we trained a model to classify variants as functional (>20% function) or LOF (<20% function). Our model outperformed existing state-of-the-art methods as evaluated by multiple performance metrics, with mean area under the receiver operating characteristic curve (AUROC) of 0.895 ± 0.025. In summary, in this study we generated a rich dataset of OCTN2 variant function and localization, revealed important disease-causing mechanisms, and improved upon machine learning–based prediction of OCTN2 variant function to aid in variant interpretation in the diagnosis and treatment of CTD.

## Significance

Interpretation of missense variants in clinically important genes is a critical challenge. Loss-of-function (LOF) variants in *SLC22A5* (OCTN2) cause Carnitine Transporter Deficiency (CTD), a rare but potentially lethal inborn error of metabolism. Motivated by the difficulty in interpreting the large number of variants of uncertain significance (VUSs) in OCTN2, we elucidated the functional effects of 150 OCTN2 missense variants and identified LOF variants in all major ancestral populations. We identified improper subcellular localization to be a major LOF mechanism for OCTN2 variants, with the potential to be targeted in novel therapies for CTD. We developed a protein-specific variant effect prediction model for OCTN2 and made functional predictions for all 10,583 missense variants to improve interpretation for clinical decision-making around CTD.

Loss-of-function (LOF) variants in transporters in the Solute Carrier (SLC) superfamily are responsible for over 100 rare genetic diseases (1, 2). Carnitine Transporter Deficiency [CTD; OMIM #212140 (3); also known as primary carnitine deficiency or carnitine uptake defect] is a rare metabolic disorder caused by biallelic LOF variants in *SLC22A5*, the gene that encodes the plasma membrane carnitine transporter OCTN2. Without timely detection, CTD can be fatal (4–6), but clinical outcomes are relatively successful when diagnosed early and treated with supplemental L-carnitine (7), highlighting the need for sensitive diagnostic practices (8).

As an actionable monogenic disorder, CTD is included in newborn screening (NBS) programs throughout the United States. Tandem mass spectrometry using samples from newborn dried blood spots is the primary screen to flag newborns with abnormally low plasma carnitine levels for further testing. However, use of biochemical assays in NBS for CTD encounters several limitations, and confirmatory diagnosis can be arduous. Biochemical assays in newborn dried blood spots result in many false-positive cases, in part because newborn carnitine levels are influenced by maternal carnitine levels, which can be low due to undiagnosed maternal carnitine deficiency or pregnancy-associated reduction in total carnitine (9). Furthermore, the plasma carnitine cutoff value for prompting further workup is not standardized: thresholds that are too high burden NBS programs with many false-positives, whereas thresholds that are too low result in false-negatives with potentially fatal consequences (9). The poor performance of biochemical-based NBS for CTD in New Zealand resulted in the discontinuation of CTD screening (10), a consideration also underway in Germany, where many cases are reportedly missed by NBS (11). Confirmatory testing for CTD following abnormal biochemical results includes sequencing of the *SLC22A5* gene. Transporter functional assay may also be performed, though this is burdensome and not timely as it requires fibroblasts cultured from a skin biopsy.

Although DNA sequencing may result in early and definitive diagnosis through the identification of variants with already known disease association, the identification of variants of uncertain significance (VUSs) and rare or novel variants with unknown clinical consequence can make diagnosis via sequencing difficult. For example, the ClinVar database (12) cataloging clinical significance of genetic variants has entries for 252 unique missense OCTN2 variants. Less than a quarter of these variants have clinical interpretations (seven variants assigned benign or likely benign, 50 variants assigned pathogenic or likely pathogenic), with the remaining 77.4% of variants classified as either conflicting interpretation (*n* = 30) or VUS (*n* = 165). Importantly, the majority of OCTN2 variants underlying CTD are missense variants (13).

The interpretation of variation in clinically important genes represents a key challenge in genomic medicine (14). While computational predictions are an important tool that can be used to aid in variant interpretation, there are several barriers to accuracy. Because the majority of prediction models are trained on large datasets of cataloged variants derived from individuals with European ancestry (15–17), an unfair bias is incorporated into the prediction methods, with decreased accuracy for variants in individuals of non-European ancestries (18). Further, highly cited gene-agnostic prediction models perform worse for membrane proteins (such as OCTN2) compared to soluble proteins (19). Recently, a number of protein-specific variant effect predictors have been successful in outperforming gene-agnostic models (20–24), though none have resolved issues of genomic inclusion.

Training computational models to perform variant effect prediction requires a large amount of data that represent the population served. Thus, the most obvious candidate proteins for such models are those that are linked to highly penetrant monogenic diseases and are easily assayable. In this study, we sought to characterize the function and localization of 150 genetic variants in OCTN2 from ancestrally diverse populations, with the ultimate goals of 1) informing inclusive diagnostics and therapeutic strategies for LOF CTD variants and 2) using machine learning to build a protein-specific model to predict the functional impact of novel OCTN2 missense variants.

## Results

### Carnitine Uptake Studies Reveal a Continuous Spectrum of Function of OCTN2 Variants.

**Fig. 1.**
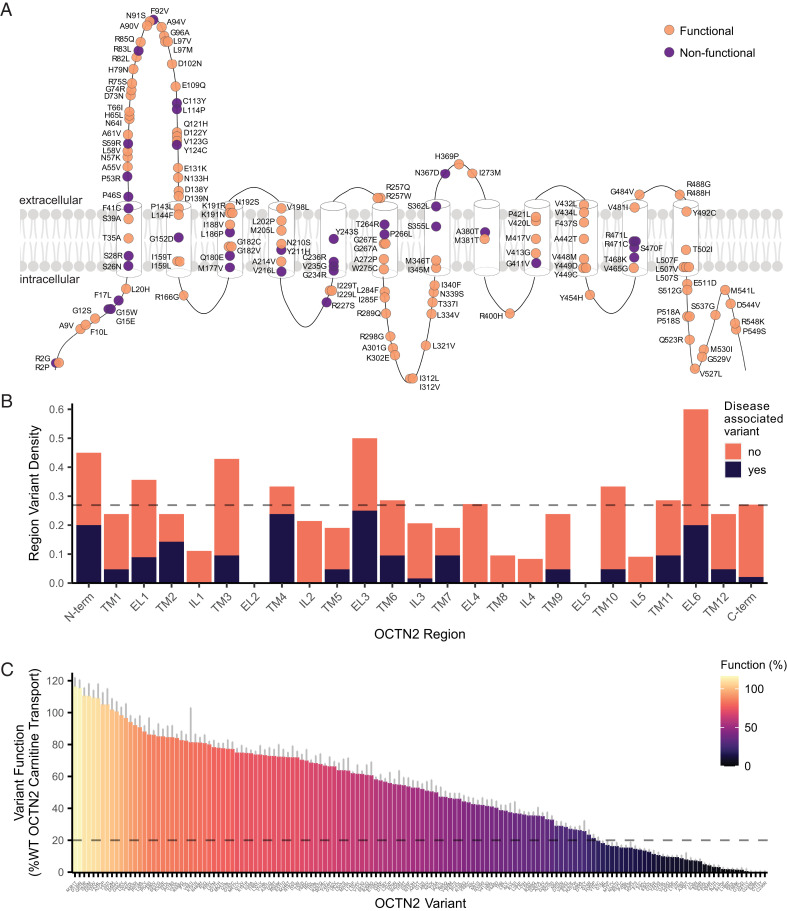
Functional characterization of OCTN2 variants. (*A*) Two-dimensional location of variants selected for characterization along the predicted secondary structure of OCTN2. Variants are colored by functional status; variants in orange are functional (>20% WT OCTN2 carnitine transport) and variants in purple are nonfunctional (<20% transport). (*B*) Density of variants characterized in each topological region of OCTN2. Variants that have been clinically associated with CTD are shown in orange, and variants that have no known clinical association are shown in purple. The dashed line represents the average density of assayed variants across all regions. N-term, N terminus; TM, transmembrane domain; EL, extracellular loop; IL, intracellular loop; C-term, C terminus. (*C*) Functional characterization of 150 OCTN2 variants with respect to ^14^C-carnitine uptake in OCTN2-expressing HEK293T cells. Each bar represents the function of an individual OCTN2 variant represented as percentage of WT OCTN2 carnitine transport. The dashed line at 20% function represents the threshold below which variants may increase susceptibility for CTD. Data represent mean ± SEM of three individual biological replicates, all performed in triplicate.

We selected a total of 150 missense variants in OCTN2 for multiparametric characterization in this study. OCTN2 contains 12 transmembrane domains, 6 extracellular loops, 5 intracellular loops, and intracellular N- and C-termini for a total of 25 topological regions (Fig. 1*A*). To ensure that variants selected for characterization had good coverage of the entire protein, we assessed the position of each variant in the membrane topology of OCTN2. Selected variants spanned the entire predicted secondary structure of the transporter and were present in each intracellular, extracellular, and transmembrane domain of the protein with the exceptions of extracellular loops 2 and 5, which contained four and three residues, respectively (Fig. 1*A*). The density of variants characterized per transporter region, calculated as the number of variants assayed in each region divided by the total number of residues in that region, ranged from 0.00 to 0.60, in comparison with the overall variant characterization density of 0.27 (Fig. 1*B*). We characterized variants both associated and unassociated with CTD. Variants associated with CTD were present in most transporter regions (Fig. 1*B*), accounted for 25.3% of assayed variants (38/150; Dataset S1), and had lower mean function than variants unassociated with CTD (*SI Appendix*, Fig. S1).

As the first level of characterization, we performed uptake studies with radiolabeled ^14^C-carnitine to determine the effect of each variant on OCTN2 function. Interestingly, we observed a continuous functional spectrum, with variant function ranging from −0.25 to 116% of the carnitine transport of the wild-type (WT) OCTN2 (Fig. 1*C*). Forty-three OCTN2 variants had no significant impact on transporter function compared to the reference OCTN2, while 107 variants had a statistically significant reduction in carnitine transport (Dataset S1). Importantly, nearly one-quarter of variants assayed (37 variants) exhibited carnitine transport reduced to less than 20% of WT function, a threshold previously demonstrated to indicate susceptibility for CTD in patient fibroblasts (13). The majority (26/37) of LOF variants are located in transmembrane domains (Fig. 1*A*). Presence of a green fluorescent protein (GFP) tag at the C terminus of OCTN2 had no effect on transporter function (*SI Appendix*, Fig. S2).

### All Ancestral Groups Harbor Variants That Exhibit a Range of Function.

**Fig. 2.**
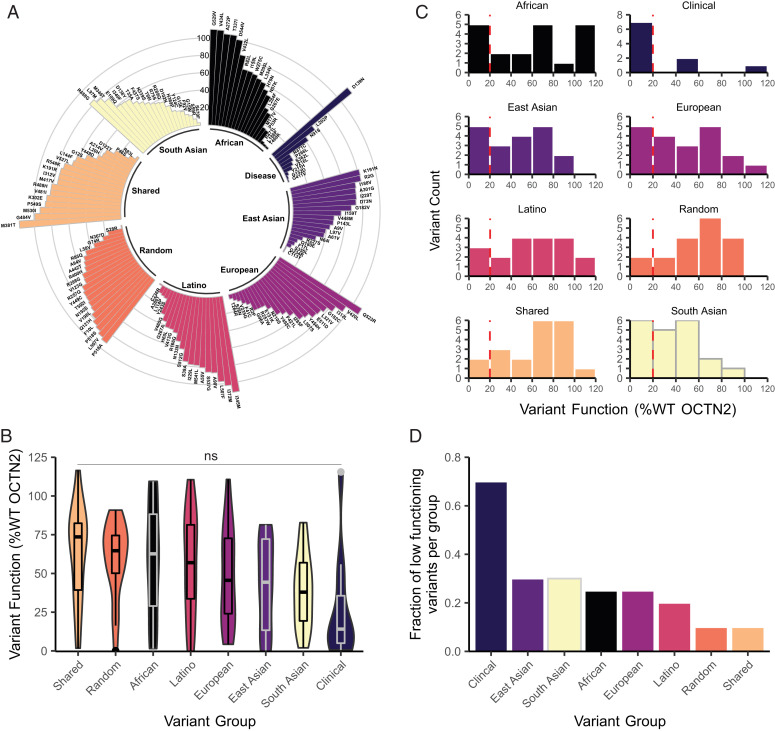
Functional distribution of variants by ancestral group. (*A*) Function of individual OCTN2 variants in each ancestral group. Height of bars (radial *y* axis) represents variant function (%WT OCTN2 carnitine transport). (*B*) Violin plots summarize the function of variants in each group. Each embedded box plot summarizes median, interquartile range, and whiskers in the style of Tukey. ns, not significant as determined by ANOVA with Tukey post hoc test. (*C*) Histogram of the distribution of variants from each group into functional bins. Vertical red dotted line illustrates the cutoff of 20% function, below which variants have increased risk for CTD. (*D*) Fraction of low-functioning variants (<20% WT) assayed in each variant group.

OCTN2 variants characterized in this study were carefully selected to ensure equal representation from diverse ancestral populations (*SI Appendix*, Fig. S3). Though we selected the most common variants in the Genome Aggregation Database (gnomAD), all variants were rare (allele frequency <0.01), and overall allele frequency had minimal association with function (*SI Appendix*, Fig. S4*)*. We included variants shared by two or more ancestral populations (“Shared”), variants exclusively found in individuals with African, East Asian, European, Latino, and South Asian ancestries, and variants selected from gnomAD at random (blinded to ancestry) and enriched the dataset with variants with known clinical associations to CTD (“Clinical”). Of note, each group harbored variants spanning a complete range of function (Fig. 2*A*). Median function of OCTN2 variants in the Shared, Random, African, Latino, European, East Asian, South Asian, and Clinical groups was 73.6, 64.7, 62.7, 57.0, 45.5, 44.4, 37.9, and 14.1% of WT function, respectively (Fig. 2*B*). Difference in mean function of variants within each group was insignificant (*P* ≥ 0.059). We next examined the number of low-functioning variants per group, defined as variants with function less than 20% of WT function. Interestingly, all groups harbored LOF variants, and Clinical variants had the largest fraction of low-functioning variants (7/10), more than double the fraction of low-functioning variants in any other group (Fig. 2 *C* and *D*), as expected.

### OCTN2 Variant Subcellular Localization Significantly Associates with Function.

**Fig. 3.**
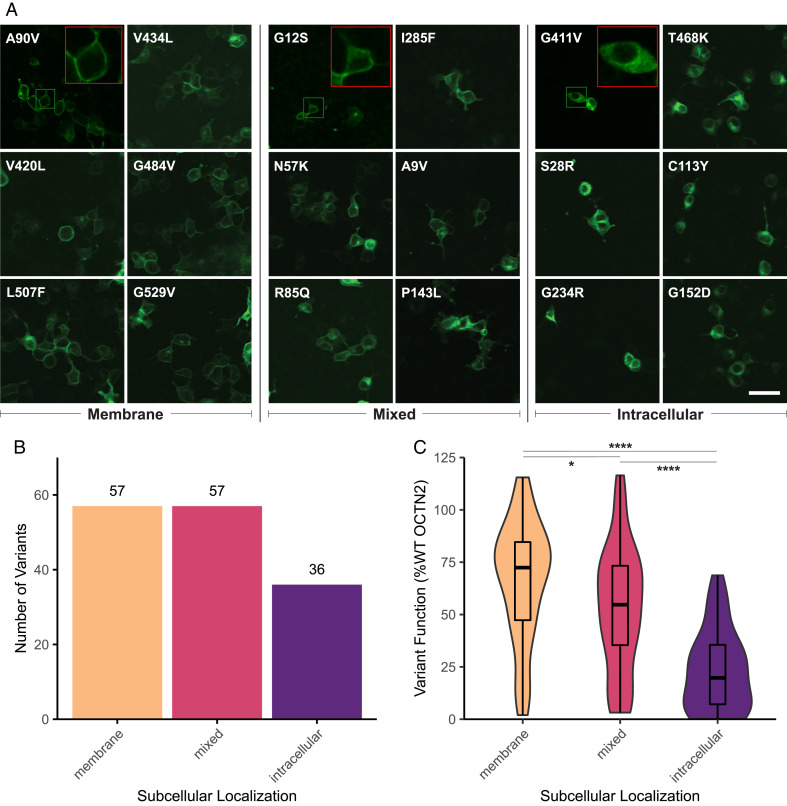
Subcellular localization of OCTN2 variants. (*A*) Representative images of OCTN2 variants conjugated to msfGFP in HEK293T cells. Three distinct patterns were observed: membrane localization (*Left*), intracellular localization (*Middle*), and mixed localization (*Right*). (Scale bar, 50 µm and is consistent for all images.) One *Inset* is shown for each localization pattern with original area outlined in green and 3× zoom in *Upper Right Corner* outlined in red. (*B*) Distribution of variants with each subcellular localization pattern. (*C*) Box plot–embedded violin plots show distribution of variant function with respect to carnitine transport based on variant subcellular localization. Box plots display median and interquartile range (IQR), and whiskers extend to the most extreme value no more than 1.5*IQR in either direction. *P value < 0.05; ****P value < 0.0001; Welch’s ANOVA between means with Games-Howell post hoc test.

To better understand the mechanisms contributing to OCTN2 LOF, we determined the subcellular localization of all 150 OCTN2 variants conjugated to monomeric superfolder green fluorescent protein (msfGFP) in human embryonic kidney cells (HEK293T) cells (25). Confocal imaging revealed that subcellular localization could be classified into three major localization patterns (Fig. 3*A* and Dataset S1); membrane localization notes the OCTN2 variant localizes primarily to the plasma membrane of the cell similar to the WT transporter, intracellular localization indicates the variant is largely retained in the cytoplasm with minimal or no presence on the plasma membrane, and mixed localization indicates the variant displays a combination of the former patterns, with partial membrane localization in combination with increased intracellular GFP intensity compared to WT (Fig. 3 *A*, *Inset* showing representative cell for each phenotype). Fifty-seven variants displayed membrane localization, 36 variants exhibited intracellular retention, and 57 variants had mixed localization (Fig. 3*B*). Subcellular localization was associated with degree of function: variants on the membrane had the highest median function (72.4% of WT OCTN2 function), and variants with mixed subcellular localization had a median function of 54.5%, whereas variants retained intracellularly had the lowest median function at 19.0% (Fig. 3*C*). A subset of variants (p.V216L, p.V235G, p.Y243S, p.S470F, and p.R471C) exhibited complete LOF despite proper membrane localization, suggesting that additional mechanisms for LOF may occur.

### OCTN2-Specific Variant Effect Prediction Models Outperform Existing Methods.

**Fig. 4.**
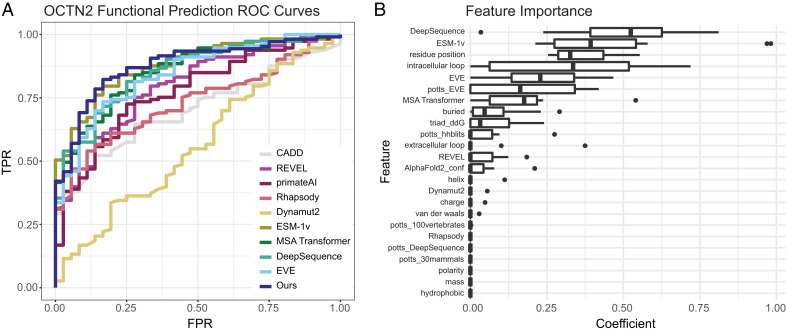
Performance of OCTN2 functional classification model. (*A*) Receiver operator characteristics (ROC) curve for our model compared to other variant effect prediction models. FPR, false-positive rate; TPR, true-positive rate. (*B*) Importance of features in performance of our model by normalized LASSO coefficient. Feature importance is represented in box plots for each feature showing the distribution of beta-coefficient from the LASSO model across each fold in 10-fold repeated random sampling. Middle bar and width of box plots represent median coefficient and interquartile range (IQR), respectively, and whiskers extend to the most extreme value no more than 1.5*IQR in either direction. Values for each feature were scaled independently from 0 to 1 prior to model training in order to make coefficients directly comparable. Features are described in depth in *SI Appendix*, *SI Text*.

Most OCTN2 variants identified in CTD patients exhibit severe LOF, with the least functional variants associating with more severe disease presentation (26). Thus, we built a classification model to predict whether OCTN2 missense variants would be LOF, defined as 20% or less than that of WT, a clinically meaningful cutoff (13). During model selection, we evaluated every combination of three types of machine learning models and four feature sets for each of the 150 OCTN2 variants (see the Machine Learning section in *Materials and Methods*). We found that a LASSO penalized logistic regression classifier achieved the best performance on the test data and literature-derived variants with mean area under the curve (AUC) beneath the receiver operating characteristic (ROC) curve of 0.90 and 0.94 for test data and literature data, respectively. We compared the performance of our model to 10 other variant prediction models: REVEL (27), primateAI (28), PolyPhen-2 (29), Rhapsody (30), CADD (31), Dynamut2 (32), ESM-1v (33), MSA Transformer (34), Deep Sequence (35), and EVE (36). We found that our model achieved the best AUC among models tested, indicating that it outperformed existing models in differentiating between functional and LOF OCTN2 variants (Fig. 4*A* and Table 1). Additionally, our model achieved an AUC of 0.95 on the functionally characterized OCTN2 variants curated from the literature (*SI Appendix*, Fig. S5*)*. We evaluated the relative importance of the input features using coefficients from the LASSO model. We found that the most important features for functional prediction were from recent state-of-the-art protein language models (e.g., DeepSequence, ESM-1v, EVE) in addition to OCTN2-specific descriptors (e.g., intracellular loop, residue position) (Fig. 4*B*).

**Table 1.** Performance of models in the classification of OCTN2 variants as LOF (<20%) or functional (>20%)

**Predictor**	**AUC**	**Accuracy**	**Sensitivity**	**Specificity**	**PPV**	**NPV**	**MCC**
Ours	0.895 ± 0.025*	0.856 ± 0.034*	0.890 ± 0.053*	0.845 ± 0.052	0.653 ± 0.079	0.964 ± 0.017*	0.673 ± 0.061*
ESM-1v	0.879	0.805	0.796	0.833	0.938*	0.566	0.563
MSA Transformer	0.854	0.772	0.761	0.806	0.925	0.518	0.501
DeepSequence	0.854	0.799	0.814	0.750	0.911	0.563	0.517
EVE	0.845	0.758	0.833	0.735	0.500	0.933	0.496
REVEL	0.797	0.745	0.722	0.752	0.481	0.895	0.422
primateAI	0.774	0.732	0.750	0.726	0.466	0.901	0.418
PolyPhen2	0.753	0.779	0.556	0.850*	0.541	0.857	0.401
Rhapsody	0.734	0.624	0.889	0.540	0.381	0.938	0.370
CADD	0.702	0.604	0.861	0.522	0.365	0.922	0.331
Dynamut2	0.563	0.405	0.336	0.806	0.844	0.279	0.132

^*^The highest performance for each metric.

In addition to the classification model that predicted binary function of variants, we trained a regression model to quantitatively predict the function of OCTN2 variants. We first performed model selection in the same way as described in the classification section, finding that the LASSO penalized linear regression model performed best. We evaluated regression model performance with an R^2^ metric, defined as the proportion of variance in measured function that is explained by predicted function. Our model had an R^2^ of 0.55, considerably higher than any of the other models evaluated in this study. For comparison, the next best performing model was ESM-1v with an R^2^ of 0.45.

**Fig. 5.**
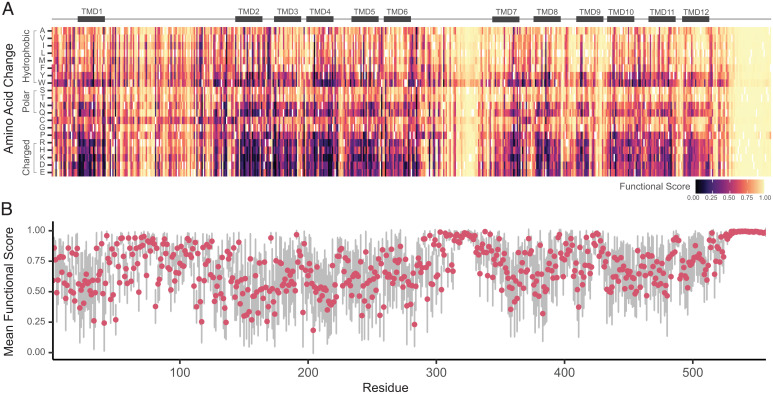
Predicted function of all possible missense variants in OCTN2. (*A*) Normalized functional score for all possible substitutions at every residue. Functional scores greater than 0.5 indicate function greater than 20% of WT OCTN2 function, with scores closer to 1 indicating increased confidence in prediction; functional scores less than 0.5 indicate function less than 20% of WT OCTN2, with scores closer to 0 indicating increased confidence in prediction. Reference residues are colored in white. Cartoon of OCTN2 secondary structure is aligned above heatmap. TMD, transmembrane domain. (*B*) Mean functional score for each residue position. Dots and bars represent mean and SD of functional score for all residues at that position, respectively.

We used our classification model to generate predictions for the function of all possible missense variants in OCTN2 (*n* = 10,583; Fig. 5 and Dataset S2). We found that 2,097 variants were predicted to cause severe LOF (<20% function), representing 19.8% of all possible variants. From these functional prediction models, we found that charged residue substitutions in transmembrane domains were predicted to be very damaging to function, whereas hydrophobic substitutions in the transmembrane domains were predicted to have minimal impact on function. Extracellular loop 1, intracellular loop 3, and the intracellular C terminus were predicted to be most tolerable to substitutions. We tested model performance on a set of seven novel variants identified in a large-scale NBS study (37) and found that the model correctly predicted the function of five variants (Dataset S3).

### Machine Learning Enables Prediction of Variant Localization.

In addition to predicting function, we trained a model to predict the effect of protein variants on subcellular localization. Informed by the subcellular localization data for all 150 OCTN2 variants obtained from confocal imaging, we aimed to predict whether proteins would be properly localized to the membrane or retained intracellularly. We trained two models: one to predict full membrane localization and a second to predict full intracellular retention. This was done because many variants presented with “mixed localization” (i.e., partial but incomplete localization to the membrane). These models attempt to predict whether a protein will have complete localization to the membrane or complete retention within the cell. A logistic regression model was able to differentiate missense variants that made it to the membrane from those that were retained intracellularly or had mixed localization with good performance (AUC: 0.74, accuracy: 0.70; *SI Appendix*, Fig. S6). Similarly, our model was able to differentiate variants that cause intracellular retention from those that have membrane or mixed localization (AUC: 0.74, accuracy: 0.78).

## Discussion

Interpretation of novel genetic variants in a clinical setting for diagnosis and treatment of genetic disorders or pharmacogenomics (PGx) remains a major challenge. In this study, we functionally characterized and determined the subcellular localization of 150 missense variants in the plasma membrane carnitine transporter, OCTN2. Our work has important implications toward improving the diagnosis and treatment of CTD. With this study of 150 missense variants, we substantially increased the number of characterized OCTN2 variants in the literature, expanding our knowledge of low-functioning at-risk variants. Importantly, we identified mislocalization as a common cause of LOF. As variant-specific treatment options become increasingly available for genetic disorders involving membrane proteins (e.g., the Cystic Fibrosis Transmembrane Conductance Regulator [CFTR]), this information has the potential to be leveraged in future therapy for individuals harboring particular variants. Finally, this wealth of data was used to build machine learning models to predict function and localization of all possible missense variants in OCTN2. The models greatly improved upon performance of current prediction algorithms for OCTN2 and provided robust predictions for all potential variants in the transporter. Below, we describe our major findings in the context of the literature.

Functional assays provide powerful tools for interpretation of genetic variants identified clinically. For example, recent studies demonstrate that in vitro functional characterization could aid in the reclassification of the majority of missense VUSs (38). Interestingly, our functional characterization of OCTN2 missense variants from the gnomAD database revealed there to be a continuous distribution of OCTN2 function (Fig. 1*C*). This is consistent with previous findings; carnitine transport assayed in fibroblasts from 358 individuals investigated for potential CTD revealed a similar functional spectrum (13). About 25% of the gnomAD variants screened were LOF, reduced carnitine transport to 20% or less than control, and in theory, have the potential to cause CTD in either individuals homozygous for the variants or in compound heterozygotes. These variants are ultra-rare and have not been observed in homozygous individuals, though we cannot exclude their presence in compound heterozygotes.

To continue to expand the number of characterized OCTN2 variants with known clinical association, we enriched the variants selected for this study with 10 additional variants found in confirmed or suspected cases of CTD (Fig. 2*A* and Dataset S4). Seven of the clinically associated variants, including all four from confirmed cases, were LOF, retaining less than 20% of WT carnitine transport. Three variants classified by ARUP as VUSs were LOF, and three variants retained partial or complete function: p.N91S, p.L202P, and p.D139N functioned at 42.3, 55.9, and 115.5% of WT, respectively (*SI Appendix*, Fig. S7), suggesting an uncertain role as determinants of low plasma carnitine levels.

Our effort to make an ethical selection of variants for study with equal representation from major ancestral groups in the gnomAD database allowed us to analyze trends in OCTN2 function across diverse populations. We found that LOF OCTN2 variants were identified in all major ancestral populations from gnomAD (Fig. 2*A*). While CTD is rare, prevalence varies globally with estimated incidences of 1:300 in the Faroe Islands (39), as high as 1:8,200 in some regions of China (40), 1:40,000 in Japan (39, 41), and up to 1:75,000 in California (9). We found no significant difference in mean variant function between groups (Fig. 2*B*). Indeed, regions with a higher incidence of CTD tend to have common founder variants affecting many individuals [e.g., p.N32S in the Faroe Islands (5) and p.R254X in China (8)], rather than an increased number of unique pathogenic variants. Reported incidence rates of diagnosed CTD are substantially lower than expected based on the population-specific allele frequencies of pathogenic variants, suggesting that NBS misses cases at an alarming rate (9–11, 13). Incidence rates of CTD have not been reported in countries with primarily African, Latino, or South Asian ancestries to our knowledge. However, based on the population-specific allele frequencies of LOF variants identified in our study alone, we estimate minimum carrier frequencies to be 1:399 in people of African ancestry, 1:193 in East Asians, 1:437 in Europeans, 1:305 in Latinos, and 1:169 in South Asians (Dataset S5).

In tissue, OCTN2 localizes to the apical membrane of enterocytes in the gut and renal proximal tubular cells in the kidney, where its major functions are to absorb and reabsorb carnitine into the systemic circulation, respectively. Here, we identified mislocalization of the carnitine transporter (Fig. 3*A*) as a common LOF mechanism (Fig. 3*C*), with 62% of variants in our study exhibiting partial or complete intracellular retention (Fig. 3*B*). This knowledge has the potential to be leveraged in novel therapeutic approaches for CTD, where we suggest that a pharmacochaperone designed to stabilize OCTN2 protein folding could rescue membrane localization and restore a degree of function to missense transporter variants. Even minimal increases in membrane localization and function could be sufficient to maintain systemic carnitine levels (42). Such therapeutic approaches have been successful for CFTR in cystic fibrosis (43), clinically tested for enzyme deficiencies (44), and explored for norepinephrine, dopamine, and serotonin transporters NET/*SLC6A2*, DAT/*SLC6A3*, and SERT/*SLC6A4* (45). In the event that a pharmacochaperone is developed for OCTN2, we envision subcellular localization data to be informative in personalized medicine to identify patients harboring mislocalized variants that may benefit from such treatment.

In addition to variants for which mislocalization appears to be the primary cause of LOF, we identified a population of variants that localized properly to the plasma membrane of the cell yet had greatly impaired function (Fig. 3*C*). Though it was not directly investigated in our study, we hypothesize that LOF in variants localizing to the plasma membrane is due to an alternative mechanism, such as disrupted transporter kinetics. Carnitine is a zwitterion, and OCTN2 is thought to have distinct carnitine and cation binding sites (46). Variants affecting these binding sites as well as those that create steric hindrance in the binding pocket or alter sodium recognition in this sodium-dependent transporter could reduce or fully prevent the binding or translocation of carnitine, increasing the K_m_ of carnitine. Notably, five of six variants (p.V216L, p.V235G, p.Y243S, p.S470F, and p.R471C, but not p.N367D) that have less than 20% function and proper membrane localization project into the translocation pore of OCTN2, based on the AlphaFold2 predicted structure (*SI Appendix*, Fig. S8). Such variants present on the membrane yet nonfunctional could, in theory, benefit from rescue by allosteric modulators.

Here, we present protein-specific variant effect prediction models trained with ethically selected variants from diverse ancestral populations. Using classification models for both function (Figs. 4 and 5) and localization (*SI Appendix*, Fig. S6), we can predict whether any possible variant in OCTN2 will be functional or will have impaired membrane localization. Despite limited experimental capacity to characterize just under 1.5% of all possible missense variants in OCTN2, our models outperform existing methods (Fig. 4*A*). Additionally, our models were trained with data equally representing diverse ancestral groups, aiming to reduce model bias and ensure comparable accuracy in the prediction of variants identified across ancestries. We identified 2,097 missense variants predicted to cause severe LOF and 578 variants predicted to be retained intracellularly, with potential for functional rescue by pharmacochaperone-based therapies. We additionally identified 1,697 variants predicted as LOF despite proper membrane localization. It should be noted that localization and function are highly correlated, as protein that does not localize to the membrane is not functional. However, we do see some differences between function and localization predictions across all 10,583 missense variants (Fig. 5 and *SI Appendix*, Fig. S6). Investigation of features most important in predicting variant function revealed that protein language models trained on millions of protein sequences were most useful to the model (Fig. 4*B*). The importance of these features suggests that evolutionary conservation is predictive of OCTN2 function. We make these predictions for all possible missense variants (Dataset S2) available for use by interested individuals, researchers, or clinicians.

The major limitation of this study is the size of the dataset, in which we functionally and spatially characterized 150 OCTN2 missense variants. While we have greatly increased the number of variants published in the literature with functional characterization, the size of the dataset limits the power to train the machine learning predictive models. Our determination of OCTN2 variant function by radioligand uptake assays allowed for sensitive detection of carnitine transport, a readout directly relevant to CTD etiology. However, the use of radioactivity is currently incompatible with deep mutational scanning (DMS) platforms that have recently increased the scale at which variants can be functionally characterized. Many DMS studies have relied on assays that are scalable yet lack direct relevance to a disease phenotype. For example, the use of fluorescence-activated cell sorting (FACS) to detect changes in the abundance of OATP1B1-GFP failed to identify variants that are expressed in the cell yet are nonfunctional (47). With functional assays that directly measure carnitine transport function, we were able to identify a larger proportion of nonfunctional variants than through fluorescence-based assays alone. Several additional limitations exist. Here, we used transient expression in HEK293T cells to measure OCTN2 variant function and determine localization. The use of this heterologous expression system is common practice in functional genomics studies of many proteins, including SLC transporters. However, it has been determined that HEK293T cells have limited transcriptomic overlap with mature kidney cell gene expression profiles (48) and instead bear more resemblance to cells from the adrenal gland. Thus, it is possible that the transporter variants behave differently in our in vitro system than in proximal tubule cells of the kidney in vivo. Limitations exist in our imaging of OCTN2-tagged variants, where we were unable to quantify colocalization between GFP (OCTN2) and the cell membrane stain due to software limitations and thus instead provided a qualitative classification of cellular membrane localization. As an alternative to high-content imaging, FACS with an antibody against an extracellular domain of OCTN2 or an inserted epitope tag (49) could be employed to determine membrane localization with more precision.

Clinical interpretation of functional genomic studies can be limited by complex genotype–phenotype relationships. For CTD, the function of a single missense variant assayed in vitro must be considered cautiously in a disease context. Conflicting reports have been published on the absence (50–52) or presence (26, 53) of a genotype–phenotype correlation. Early reports indicated that patients, and in some instances siblings, with the same OCTN2 variants had variability in symptoms, severity, and age of onset (50–52). In contrast, another study found that symptomatic patients had more nonfunctional variants, namely nonsense and frameshift (26). Finally, a study in patients from the Faroe Islands revealed a significant correlation between residual OCTN2 transporter function and plasma carnitine levels (53). Our manual literature curation of CTD patients revealed that more than 92% of patients harbor biallelic variants with an average of less than 20% function (when two variants are identified and functional data are available for both variants; *SI Appendix*, Fig. S9). Thus, interpretation of an individual’s set of variants in a clinical setting must cautiously be made by healthcare professionals in accordance with guidelines by the American College of Medical Genetics (ACMG) (14).

At a patient and community utility level, we recognize the importance of meaningful and respectful translation of sequencing results. For families navigating CTD in young children, there may be divergent views about return of results, result actionability, informed consent, and the sufficiency of parental assent for deposition and research use of variant information (54–57). It is desirable to establish patient stakeholder support in the management of and democratization of data sharing (58, 59). Nonetheless, the community benefits of characterizing variants from publicly shared databases, as this study reveals, arguably outweigh risks of not being able to achieve full informed consent for (deidentified) data use.

Ultimately, the purpose of OCTN2-specific variant effect prediction models is to inform clinical diagnostics and decision making, including therapeutic decisions. OCTN2 serves as a unique link between two major fields for which computational interpretation of genetic variants is increasingly needed: inborn errors of metabolism (IEMs) and PGx. The SLC superfamily consists of over 400 transporters, more than 100 of which are linked to IEMs and other Mendelian disorders (1, 2). Encoded by *SLC22A5*, OCTN2 also shares homology with several pharmacogenes in the SLC22 family, namely *SLC22A1* (OCT1), *SLC22A2* (OCT2), *SLC22A6* (OAT1), and *SLC22A8* (OAT3). Functional data are limited for most of these transporters, impeding the interpretation of genetic variation in IEM and PGx. Transfer learning offers a solution whereby algorithms optimized with substantial data from one protein (e.g., OCTN2) could be refined with minimal data for related proteins implicated in IEM or PGx, producing better variant interpretation predictions than would be possible alone (17).

In conclusion, here we present comprehensive functional annotation of the largest set known to date (*n* = 150) of missense variants in OCTN2 from the gnomAD database. We show that LOF for many OCTN2 variants can be attributed to failure to traffic to the plasma membrane, revealing a disease-causing mechanism with the potential to be leveraged in therapeutic strategies for the treatment of CTD. Further studies are ongoing to determine the mechanisms for improper sorting of variants to the plasma membrane, which may be leveraged for future therapies. The results of our protein-specific variant effect prediction model for OCTN2, with which we predict the function and localization of OCTN2 variants, substantially outperformed existing methods. We provide these functional predictions for all possible missense variants in OCTN2 (*n* = 10,583), which may be useful in clinical interpretation of novel VUSs in accordance with ACMG guidelines.

## Materials and Methods

### Variant Selection and Annotation.

Variants included in this study were carefully selected to ensure equal representation from diverse ancestral populations available in the Broad Institute’s gnomAD database (v2.1.1) (60). A total of 150 *SLC22A5* missense variants were selected for characterization (for simplicity, referred to as OCTN2 variants to signify the change at the protein level). Variants assigned to each ancestral population as defined by gnomAD (African, East Asian, European, Latino, South Asian) were exclusive to that population (Dataset S6). Detailed workflow for the selection of OCTN2 variants characterized in this study can be found in *SI Appendix*, Fig. S3. Predicted membrane topology of OCTN2 was modeled from UniProtKB (#O76082). Clinical association of all variants was annotated based on literature and database review and identification of that variant in an individual clinically diagnosed with CTD or suspected of possible CTD due to low carnitine levels and presence of at least one variant in OCTN2. Search terms for literature review included “SLC22A5 mutation,” “OCTN2 mutation,” “primary carnitine deficiency case study,” and “primary carnitine deficiency newborn screening.” We compiled a list of previously characterized variants published in the literature to use as an additional dataset for performance evaluation of our machine learning models. Variants included in the dataset met the following criteria: 1) the variant was expressed and assayed in a mammalian system, and 2) the experiment measured the carnitine transport of a single missense OCTN2 variant. The dataset is referred to as “Literature Variants” (Dataset S7).

### Cell Culture.

HEK293T cells were cultured in Dulbecco’s modified Eagle medium (Life Technologies) supplemented with 10% fetal bovine serum (GE Healthcare Life Sciences) and penicillin/streptomycin (100 U/mL) (Life Technologies) and grown in a humidified incubator at 37 °C with 5.0% CO_2_.

### Construct Generation.

A custom WT OCTN2 plasmid was generated by golden gate cloning as previously described (61). Full-length *SLC22A5* complementary DNA (cDNA) (NM_003060.4) was synthesized (Twist Bioscience) with the start codon removed and adapter sequences synthesized on either end to facilitate golden gate cloning reactions. The linear *SLC22A5* cDNA was domesticated into the MTK0_027 entry vector by golden gate cloning with BsmBI. A second golden gate cloning reaction was performed with BsaI to assemble the transcription unit (TU) plasmid, which included parts MTK1_001, MTK2_023, MTK3a_030, the MTK0_027-SLC22A5 as part 3b, MTK4a_015, MTK4b_001, MTK5_006, and MTK678_001. Description of parts is provided in *SI Appendix*, Table S1. A third golden gate cloning reaction was performed with BsmBI to insert the *SLC22A5* TU into the MTK0_017 destination vector. MTK plasmids detailed in the Construct Generation section below were a generous gift from the Hana El-Samad Lab (University of California, San Francisco [UCSF], San Francisco, CA). Assembled constructs were sequenced (MCLAB, South San Francisco, CA) to validate successful cloning and ensure absence of mutations. All OCTN2 variants selected for functional characterization were synthesized by site-directed mutagenesis (Genscript) from the final assembled *SLC22A5* construct.

### Transient Transfection of Plasmids Containing OCTN2 Variants.

HEK293T cells containing a landing pad at the hAAVS1 locus were used to create transient or stable cell lines (61). Transient transfection of constructs encoding the WT OCTN2 and OCTN2 variants was achieved by reverse transfection using Lipofectamine LTX transfection reagent (Thermo Fisher Scientific) according to manufacturer’s protocol. The MTK0_017 destination vector construct was used as the empty vector. Constructs were mixed with Lipofectamine LTX in OptiMEM media (Life Technologies), vortexed for 10 s, allowed to stand at room temperature for 15 min, and then added to poly-D-lysine–coated 96-well plates. Each well received 100 ng of DNA and 0.2 µL Lipofectamine LTX. HEK293T cells were counted and seeded into wells at a density of 35,000 cells/well. After transient transfection, cells were cultured for an additional 48 h before subsequent experiments were performed (uptake assays or confocal imaging).

### In vitro Uptake Assays.

Forty-eight hours after reverse transient transfection of OCTN2 variants in poly-D-lysine–coated 96-well plates (Thermo Fisher Scientific, #356461), culture medium was removed, and cells were washed three times with Hank’s buffered salt solution (HBSS) (Life Technologies) at 37 °C and preincubated with the third wash of HBSS for 10 min at 37 °C. Eighty microliters of 1 µM ^14^C-L-carnitine hydrochloride (Moravek Biochemicals, #MC1147) in HBSS (reaction mix) was added to each well and incubated at 37 °C for 10 min, a time point within the linear uptake phase of OCTN2 (62). After 10 min, the reaction mix was aspirated and the cells were washed 3x with ice-cold HBSS; 280 µL lysis buffer (0.1 N NaOH, 0.1% vol/vol sodium dodecyl sulfate) was added to each well, and cells were lysed on an orbital shaker for 1 h. Then, 230 µL cell lysate was removed from each well and added to liquid scintillation tubes with 2.5 mL Ecolite Liquid Scintillation Mixture (MP Biomedicals, #0188247501). Tubes were vortexed, and the radioactivity in each sample was measured on a Beckman LS6500 liquid scintillation counter (Beckman Coulter). Twenty-five microliters of lysate from each well was reserved for protein concentration determination by Pierce BCA Assay. Function of each variant was normalized to WT OCTN2 and expressed as a percentage after background carnitine uptake measured in the empty vector (EV) was subtracted from both, calculated as follows: (Variant – EV)/(WT – EV)*100. Each variant was assayed in triplicate on a 96-well plate and measured in three biological replicates.

### Confocal Imaging and Localization Classification.

Plasmids encoding OCTN2 variants with a C-terminal monomeric msfGFP tag were transiently transfected into HEK293T cells seeded at 20,000 cells/well in black wall poly-D-lysine–coated 96-well plates (Greiner Bio-One, #655946) as detailed above. One variant was transfected per well. After 48 h, the plasma membrane was stained for 5 min with Wheat Germ Agglutinin Alexa Fluor 647 Conjugate (Thermo Fisher Scientific) diluted 1:500 in HBSS, and cells were fixed with 3.7% formaldehyde in HBSS for 20 min. Nuclei were stained with 10 µM Hoechst 33342 dye in HBSS (Thermo Fisher Scientific, #62249) for 20 min at room temperature. Plates were imaged with the IN Cell Analyzer 6500 confocal high-content imaging system (General Electric Life Sciences/Cytiva), using a 488-nm excitation laser. Nine images were taken per well with all samples imaged on the same day using the same image acquisition settings, and results were then replicated on two independent days. Three independent researchers reviewed images for localization and qualitatively classified variants into membrane, intracellular, or mixed categories. Reviewers were given representative images for each of the localization categories to inform their baseline understanding of categorization. All images were displayed using the same brightness and contrast values before being given to the reviewers to ensure assessment consistency. Researchers were blinded to variant name or function. The concordance between classifications by image reviewer was strong (*SI Appendix*, Fig. S10).

### Machine Learning.

Methods for generation of features used in machine learning are described in *SI Appendix*, *Methods*. Classification models were trained to predict severe LOF variants with function less than 20% of WT OCTN2 function with respect to carnitine transport. For the classification model, three types of models and four feature sets were evaluated. The three types of machine learning models were LASSO penalized logistic regression, random forest, and gradient boosting machines. Four sets of features were generated: 1) sequence-based features describing the resulting amino acid change and position in OCTN2 protein sequence; 2) structure-based features extracted from the AlphaFold-2 structural model [default model download from the AlphaFold Protein Structure Database (63, 64)]; 3) prediction-based features derived from unsupervised variant effect prediction models, including variational autoencoders (35), Potts models (65), and protein language models (33); and 4) all features combined. Features in each set are provided in *SI Appendix*, Table S2. A final classification model was trained using a subset of features that were available for every possible amino acid change. Every combination of model type and feature sets was evaluated by training through 100 iterations of random subsampling of the 150 characterized OCTN2 variants using an 80/20 train/test split. In addition to the test set, we evaluated the predictive performance of each model on a set of 82 characterized OCTN2 variants derived from the literature that were not characterized in our study (Dataset S7).

We trained and evaluated the OCTN2 function classifier by training a LASSO penalized logistic regression model using repeated random sampling using all features. We used random splits of the characterized variants with 105 variants used for training and the remaining 45 used for testing in each fold. The models were trained in R using the package Caret with repeated cross validation for hyperparameter tuning. We defined a binarizing cutoff for our model output by maximizing the sum of the sensitivity and specificity over all possible cutoffs. We then used this cutoff to calculate other metrics (e.g., accuracy) in the test set as well as the literature-derived variants. The trained model was then used to predict the function of all possible missense variants in OCTN2, including the variants in the test set and those in the literature-derived set. We evaluated the relative importance of the input features using coefficients output by the LASSO model.

We additionally trained a regression model to quantitatively predict the measured function of OCTN2 variants, with model selection performed in the same way described in the classification section; however instead of models tuned for classification, we trained models for regression. The final model was trained with 110 variants and tested with the remaining 40. LASSO coefficients were again used to evaluate feature importance.

### Data Analysis.

Statistical analysis was performed in R version 3.6.3 (R Core Team, 2020). Plots were generated using R package ggplot2 version 3.3.5. Additional figures were generated with Biorender.

For carnitine uptake assays, data are expressed as mean ± SEM (SEM) with significance determined by Student’s *t* test. A Bonferroni correction was used to adjust the significance level to α = 0.05/150 = 3.3*10^−4^. ANOVA was used to determine significance of difference in mean function by variant group. Despite a significant *P* value of 0.0234 in the ANOVA, Tukey’s post hoc test revealed there was no significant difference between any of the groups (lowest *P* value was 0.059 between Shared and Clinical groups). Difference in mean function by subcellular localization was determined by Welch’s ANOVA with Games-Howell post hoc test (rstatix package in R) due to significant differences in variance.

Multiple metrics were calculated to evaluate the performance of our classification models as well as other published models, including sensitivity or true positive rate, specificity or true negative rate, precision or positive predictive value (PPV), negative predictive value (NPV), accuracy, AUC, and Matthew’s correlation coefficient (MCC), all of which have been defined previously (20, 23).

Portions of this work were developed from the doctoral dissertation of M.L.K. (66).

## Supplementary Material

Supplementary File

Supplementary File

## Data Availability

Functional data have been deposited in ClinVar under SUB12121751, (67), and are available in Dataset S1. All study data are included in the article and/or supporting information.
