# Supplementary material for: Functional genomics of OCTN2 variants informs protein-specific variant effect predictor for Carnitine Transporter Deficiency
Source: Proc Natl Acad Sci U S A. 2022 Nov 7;119(46):e2210247119. doi: 10.1073/pnas.2210247119 (PMC9674959; doi:10.1073/pnas.2210247119)
Supplement: Supplementary File [file pnas.2210247119.sapp.pdf]

PNAS

Supplementary Information for

Functional genomics of OCTN2 variants informs protein-specific variant effect predictor for Carnitine Transporter Deficiency

Megan L Koleske, Gregory McInnes, Julia EH Brown, Neil Thomas, Keino Hutchinson, Marcus Y Chin, Antione Koehl, Michelle R Arkin, Avner Schlessinger, Renata C Gallagher, Yun S Song, Russ B Altman, Kathleen M Giacomini*

Corresponding author: Kathleen M Giacomini
Email: kathy.giacomini@ucsf.edu

This PDF file includes:

Supplementary text
Figures S1 to S10
Tables S1 to S2
Legends for Datasets S1 to S7
SI References

Other supplementary materials for this manuscript include the following:

Datasets S1 to S7

Supplementary Text

Methods

Feature Generation

Allele frequency was calculated from gnomAD v2.1.1 (1). Pathogenicity scores for all assayed OCTN2 variants were obtained from PolyPhen-2 (2), CADD (3), REVEL (4), PrimateAI (5). As no crystal structure has been solved for OCTN2, we used the AlphaFold2 predicted structure (Entry O76082) (6) for generation of structural features. Predicted stability change ($\Delta\Delta G^{stability}$) was generated with DynaMut2 (7). Solvent accessibility was generated using GETAREA (8). Prediction of variant effect on protein dynamics was generated using Rhapsody (9). Network centrality analysis (centrality degree, centrality cluster coefficient, centrality closeness, centrality betweenness, eigenvector centrality, average neighbor degree) was generated with Network Analysis of Protein Structure (NAPS) (10). Generation of other features is described below.

pLDDT and triad_ddG scores

The AlphaFold 2 predicted structure of human SLC22A5 was obtained from the AlphaFold (6) protein structure database. pLDDT scores that represent model confidence for each position in the structure were extracted as a set of features. pLDDT is continuously valued between 0 and 100, and is a prediction of the lDDT-Ca score (11) that is used to compare two models by reporting on distances between their Ca atoms at equivalent positions. The plDDT score reported by AlphaFold is a learned prediction that was calibrated using distance from the ground-truth structure during model training.

The AlphaFold 2-predicted model was then directly used as input for ddG calculations using Triad Protabit design software (https://triad.protabit.com). The structure was first standardized using the Standardize Structure App within Triad, and then single mutant stability scores (ddG) were calculated with default parameters (float distance = 7Å and backbone only scoring = off)

using the Rosetta scoring function. These parameters ensure that local interactions around the mutation site are repacked using rosetta prior to delta G calculation.

Modeling variant effects with models of evolutionary data

In order to predict the effects of mutations, we utilized conservation information derived from evolutionary homologs of the transporter SLC22A5. The state-of-the-art unsupervised variant effect predictors (that is, predictors that have not been trained with any variant screening data) fit a statistical model on a set of related, functional sequences. These models can take the form of protein language models (12), variational autoencoders (VAEs) (13) and Potts models (14). The likelihood of a sequence under the derived statistical models has been shown to correlate well with the probability that the sequence is functional.

The features of our ensemble model include previously published methods for variant effect prediction and additional features, some of which we designed ourselves. The pre-existing methods for variant effect prediction that we used are DeepSequence (13), EVE (15), ESM-1v (12), and MSA Transformer (16). DeepSequence, EVE, and MSA Transformer require an MSA to make predictions, while ESM-1v requires only a sequence. DeepSequence and MSA Transformer features were constructed using the "DeepSequence" from "Constructing Alignments". EVE features use EVE alignments.

Constructing alignments

To combine the advantages of alignments that represent different evolutionary timescales, we used 5 different alignments for downstream training. We combined deep, diverged alignments (Alignments 1, 2, 3), which we hypothesized would include coarse-grained fold information, with more alignments that sample more closely-related organisms (Alignments 4, 5).

1. HHblits: To produce this alignment, we reimplemented the alignment generation procedure described in (17). Our implementation can be run using the `mogwai-align` command in https://github.com/nickbhat/mogwai. We used UniprotKB O76082 as the

query sequence. HHblits performed 1 iteration searching against Uniclust30 with an e-value 1e-80 to produce an alignment of 7201 sequences.

2. EVE: The EVE alignment was downloaded directly from https://evemodel.org/download/protein/S22A5_HUMAN.

3. Deepsequence: We follow the approach in (12, 14) to form a deep alignment. The query sequence was searched against the UniRef100 database using the profile HMM homology search tool jackhmmer (18). Non-redundant sequences were kept with a 0.8 sequence similarity threshold. This resulted in an alignment with 7205 sequences. The alignment script can be found at https://github.com/rmrao/DeepSequence/blob/master/align.py

4. 100 vertebrates: FASTA alignments for coding regions of the UCSC Known Genes corresponding to the human reference genome (hg38/GRCh38, Feb. 2009) aligned to 100 vertebrate genome assemblies as described in http://genomewiki.ucsc.edu/index.php/Hg38_100-way_conservation_alignment were downloaded from http://hgdownload.soe.ucsc.edu/goldenPath/hg38/multiz100way/alignments/knownCanonical.multiz100way.protAA.fa.gz and subset to those fasta records with the header prefix corresponding to the ENSEMBL transcript name for SLC22A5, "ENST00000245407."

5. 30 mammals: FASTA alignments for coding regions of the UCSC Known Genes corresponding to the human reference genome (hg38/GRCh38, Feb. 2009) aligned to 30 mammalian genome assemblies as described in http://genomewiki.ucsc.edu/index.php/Hg38_30-way_conservation_alignment were downloaded from http://hgdownload.soe.ucsc.edu/goldenPath/hg38/multiz30way/alignments/knownCanonical.multiz30way.protAA.fa.gz and subset to those fasta records with the header

prefix corresponding to the ENSEMBL transcript name for SLC22A5, "ENST00000245407."

All of these alignments can be found at https://github.com/songlab-cal/slc22a5.

Modeling variant effects with Potts models

A Potts model is an undirected Markov Random Field model which has been shown to capture information about protein structure (19, 20) as well as protein function (14, 21). Adding the unsupervised likelihood as a feature has been shown to improve the performance of regression models used to predict protein function (22, 23).

In the Potts model, which models marginal effects and pairwise interactions, the likelihood of a sequence x is given by:

$$E(x) = \sum_i h_i(x(i)) + \sum_{i<j} J_{ij}(x(i), x(j))$$

$$\mathcal{L}(x) = \frac{1}{\mathcal{Z}} \exp\left(-E(x)\right)$$

Where Z is the partition function.

To use a Potts model to predict variant effects, we compute the energy difference between the variant and the wildtype reference sequence, in this case the human reference gene SLC22A5.

$$\Delta E(x) = E(x) - E(x_0)$$

Note that this variant effect can be computed without computing the partition function Z.

Separate Potts models were fit on each of the 5 alignments described in "Constructing Alignments." Each model was used separately to predict variant effects by computing the energy difference between the variant and the wildtype reference sequence.

Fitting the Potts model

Due to the combinatorial complexity of computing the partition function Z, we cannot maximize the true likelihood of the sequences. Instead, we estimate the coupling parameters J and the marginal effects h to maximize the *pseudolikelihood*, which follows the established approach in (14, 21, 24). For the optimization, we use a modified version of Adam (25) which ties together all squared updates. The Potts model implementation we used can be found in our open source MRF library https://github.com/nickbhat/mogwai. All Potts models were trained on a single NVIDIA RTX 2080 Ti GPU for 5000 gradient update steps, with a batch size of 4096 sequences and a learning rate of 0.5.

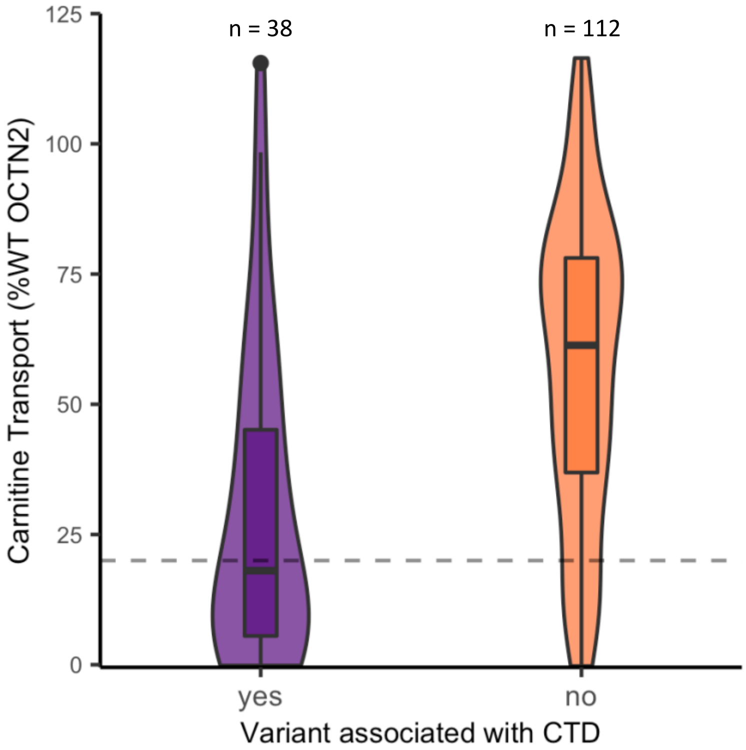

Figure S1. Variant function by clinical association. Variants identified in CTD patients have lower mean function than variants not previously identified in individuals with CTD.

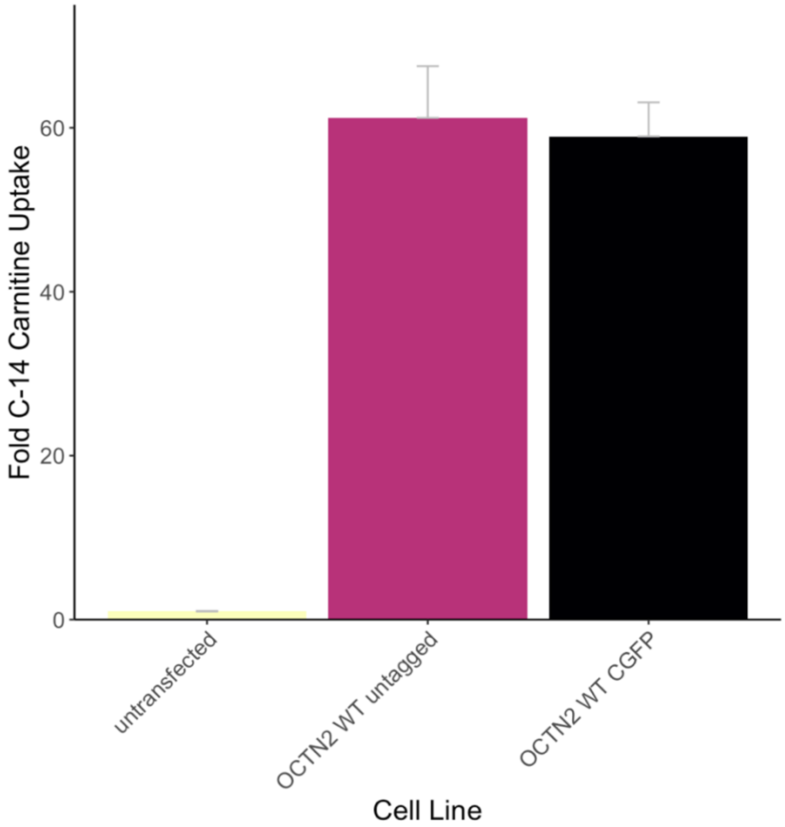

Figure S2. Effect of GFP tag on OCTN2 function. HEK293T cell lines stably expressing untagged wild-type OCTN2 and GFP-tagged wild-type OCTN2 with GFP at the C-terminus (CGFP) were created to examine the functional impact of the presence of GFP on carnitine transport. Carnitine uptake is reported as fold over untransfected cells. Data are expressed as mean + SEM from n=4. There is no significant difference between fold uptake in cells expressing untagged compared to CGFP tagged OCTN2 ($p>0.05$, Student's t-test).

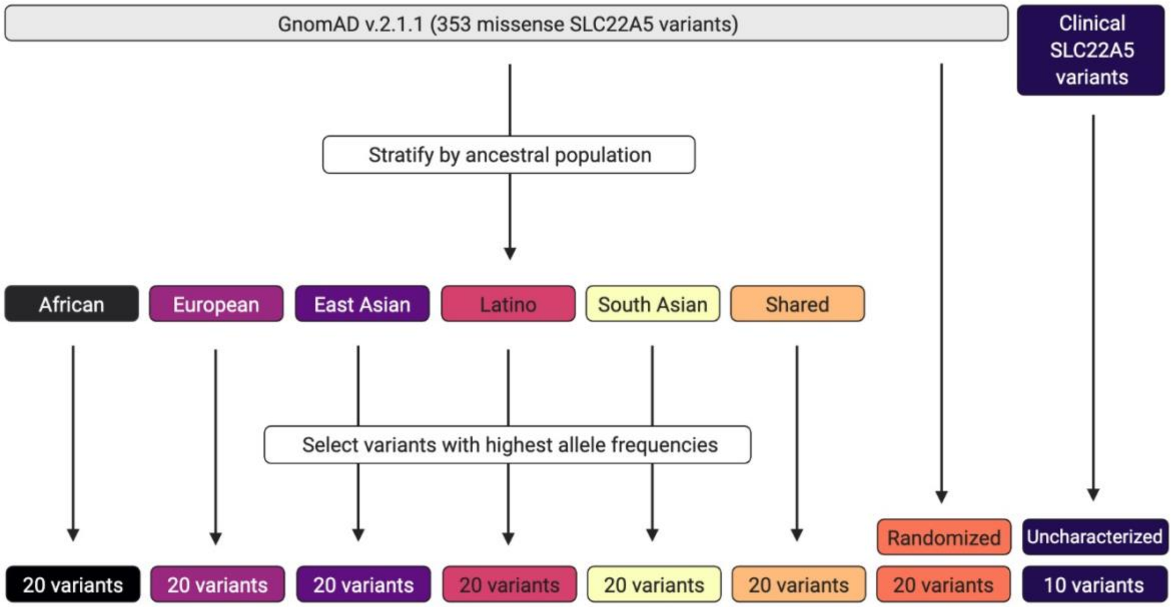

Figure S3. Workflow for selection of OCTN2 variants characterized in this study. First, gnomAD variants were stratified by ancestral population in which they were identified/classified. The top 20 population-specific variants by allele frequency were selected from each of the African, Latino, East Asian, European, and South Asian populations and were exclusive to that ancestral population (i.e., not found in any other population). Twenty additional variants were selected from the "Shared" group, defined as found in at least two gnomAD populations listed above. In addition, 20 variants were selected at random from the remaining gnomAD OCTN2 missense variants, irrespective of ancestry. Finally, 10 uncharacterized variants clinically associated with diagnosed or suspected CTD were included for study.

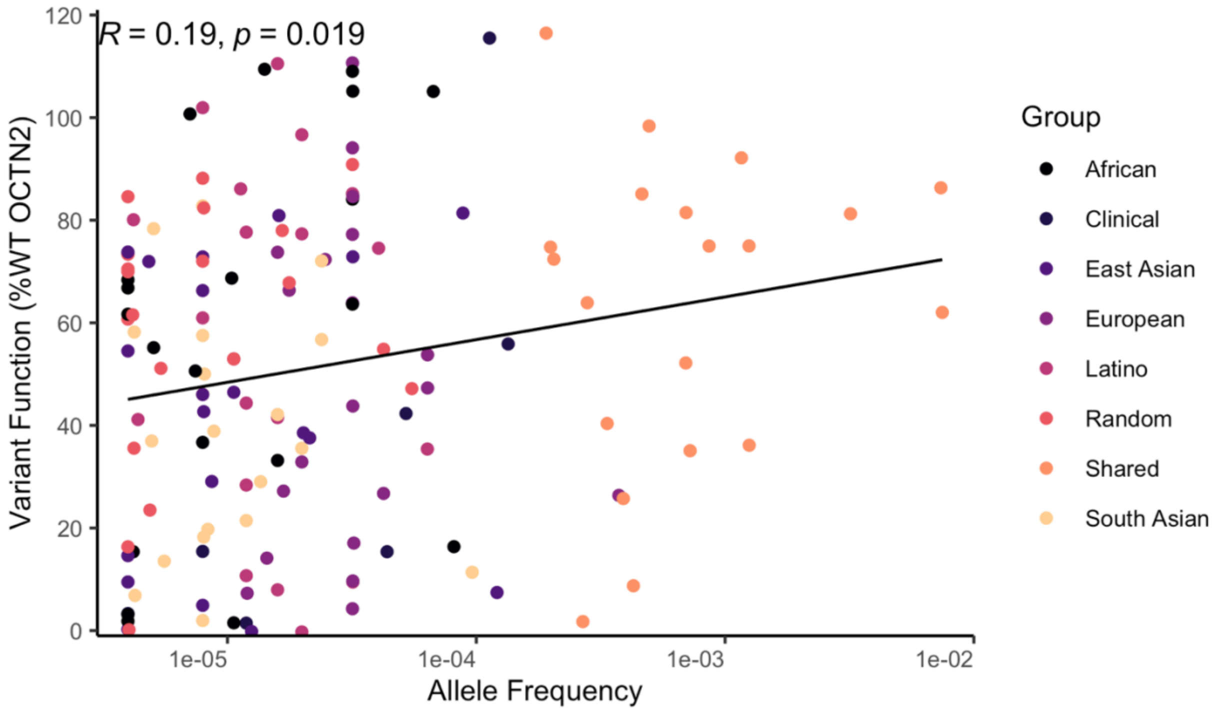

Figure S4. Variant function by global allele frequency. Global allele frequencies were obtained from the gnomAD database.

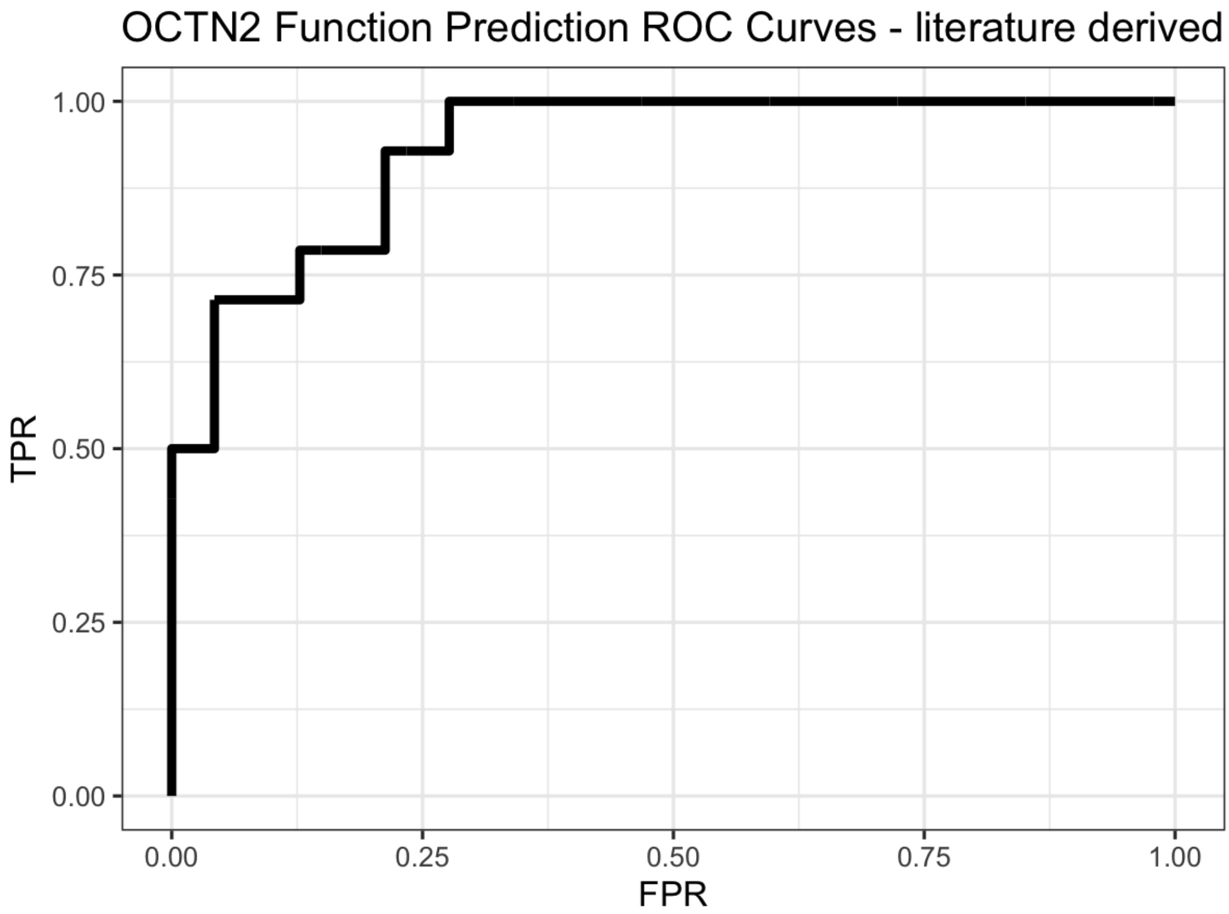

Figure S5. Validation of the LASSO penalized logistic regression classification model in predicting function of literature-derived variants independent from our study. Receiver operating characteristic (ROC) curve compares false positive rate (FPR) and true positive rate (TPR) for 82 OCTN2 variants with function derived from the literature listed in *SI Appendix, Dataset S7*. Area under the ROC curve (AUC) = 0.95.

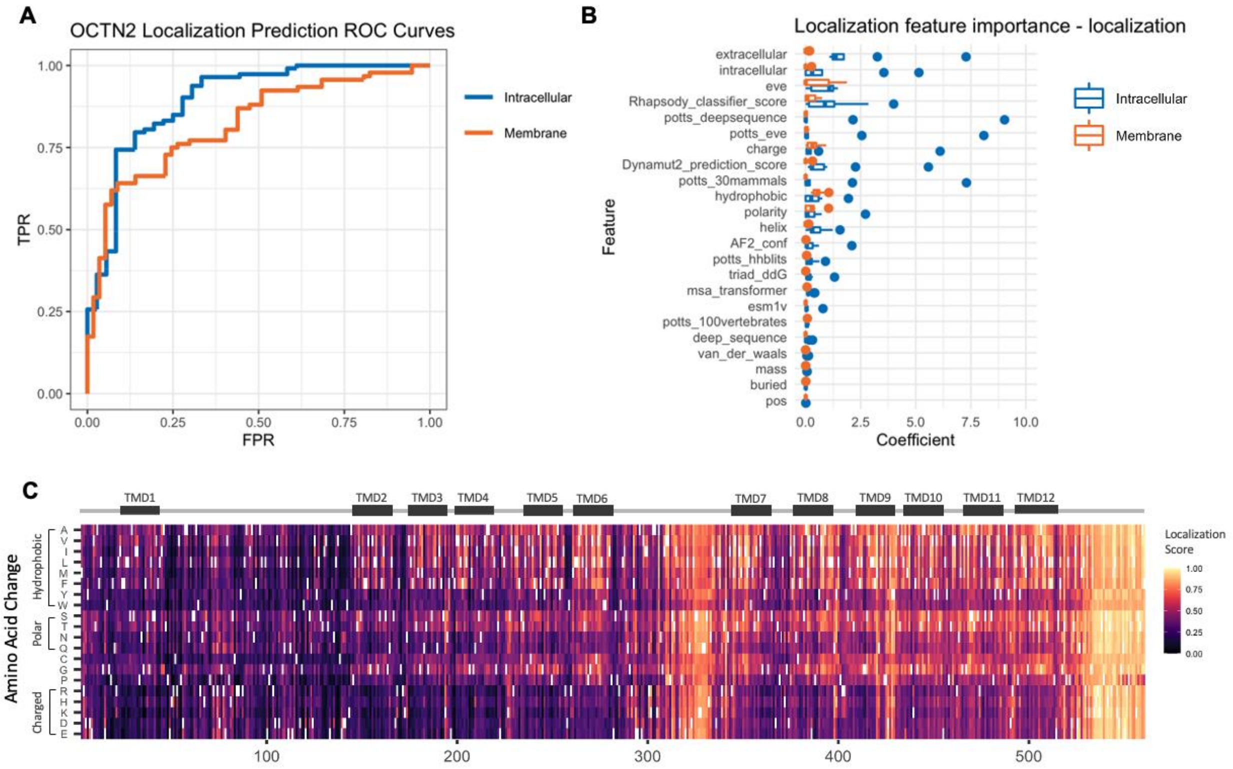

Figure S6. Performance of machine learning models to predict OCTN2 localization. (A) Receiver operator characteristics (ROC) curve for our models. Intracellular classification notes variants predicted to have intracellular localization vs membrane or mixed localization; membrane classification notes variants predicted to have membrane localization vs intracellular or mixed localization. (B) Importance of features in performance of the intracellular and membrane classification models. (C) Normalized localization score for all possible substitutions at every residue. Localization scores greater than 0.5 indicate predicted membrane localization, with scores closer to 1 indicating increased confidence in prediction; functional scores less than 0.5 indicate predicted intracellular localization, with scores closer to 0 indicating increased confidence in prediction. Reference residues are colored in white.

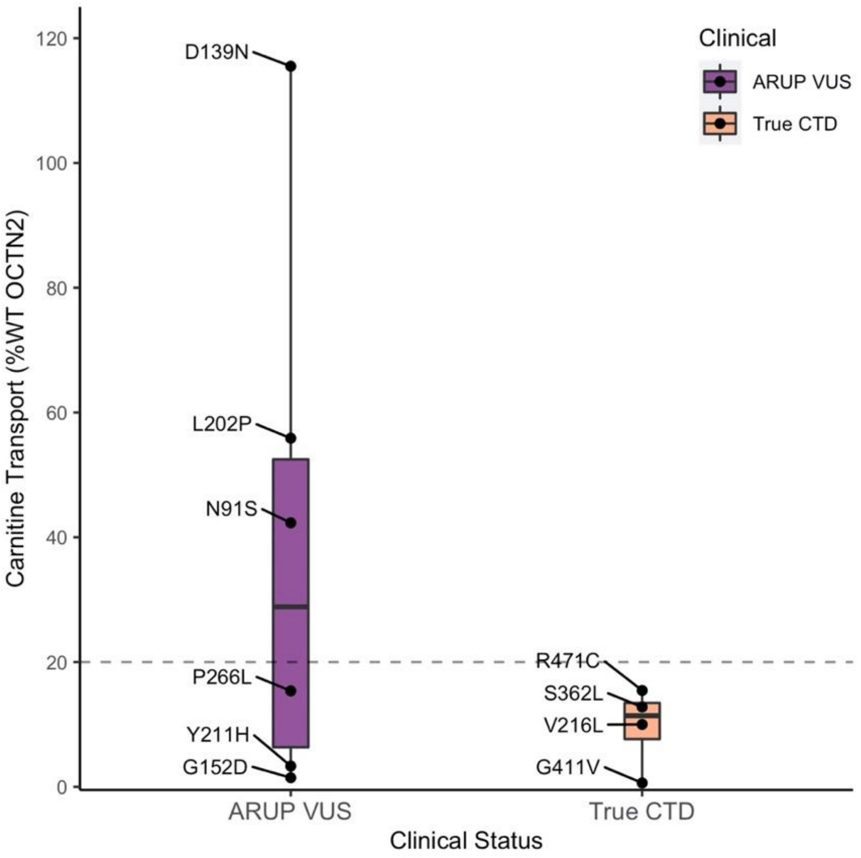

Figure S7. Function of the additional "clinical" variants added to the study in addition to the 140 variants selected from gnomAD. Variants identified in true confirmed cases of CTD are in orange, and variants in suspected cases in the ARUP database currently classified as variants of unknown significance (VUS) are in purple. The dashed line at 20% carnitine transport represents the threshold below which variants increase risk for CTD.

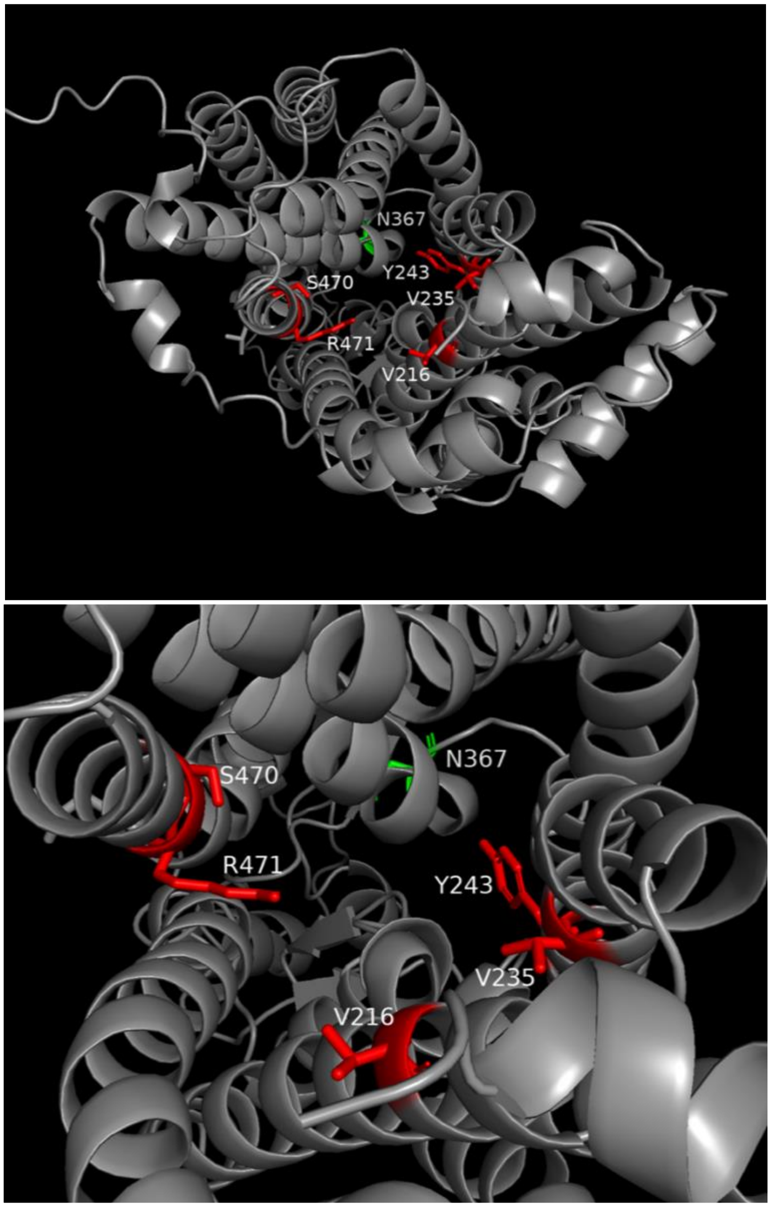

Figure S8. Visualization of properly localized, loss-of-function OCTN2 variants in the AlphaFold2 predicted structure of OCTN2. The six variants highlighted localize to the plasma membrane of the cell yet retain less than 20% function. Predicted structure suggests the variants in red project into the translocation pore of OCTN2 and may affect carnitine affinity and/or translocation. The variant in green does not appear to project into the pore. Top image is the view of the entire protein from the extracellular side, bottom image is zoomed in view from the extracellular side of the protein.

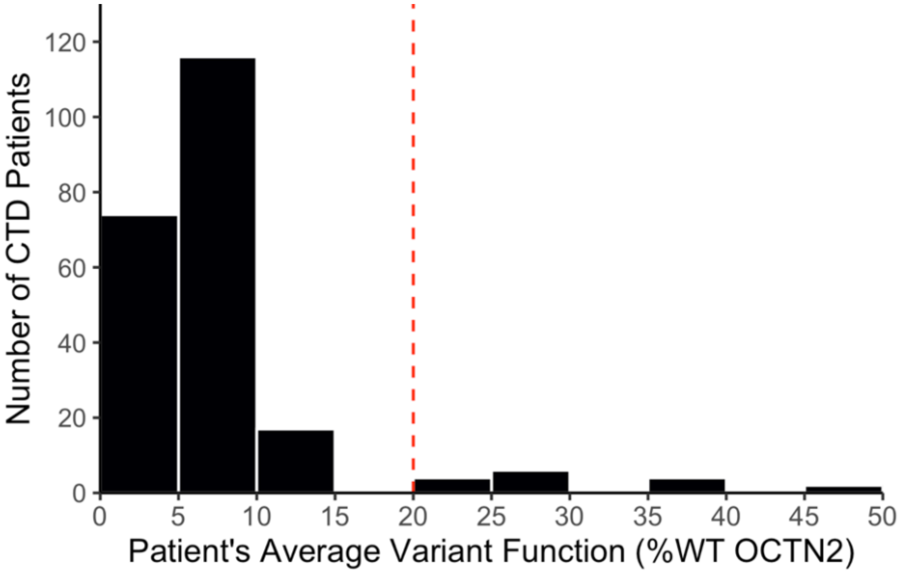

Figure S9. Average function of homozygous or compound heterozygous OCTN2 variants identified in patients with CTD. Patients with two reported variants were curated from the literature. Patients were included in analysis if quantitative function was available for both variants. Function of missense variants was derived from this study or previous measurements in the literature; nonsense and frameshift variants were presumed to have 0% function. Out of 223 CTD patients harboring two OCTN2 variants with quantified function, 207 patients (92.3%) had variants averaging less than 20% function compared to wild-type OCTN2.

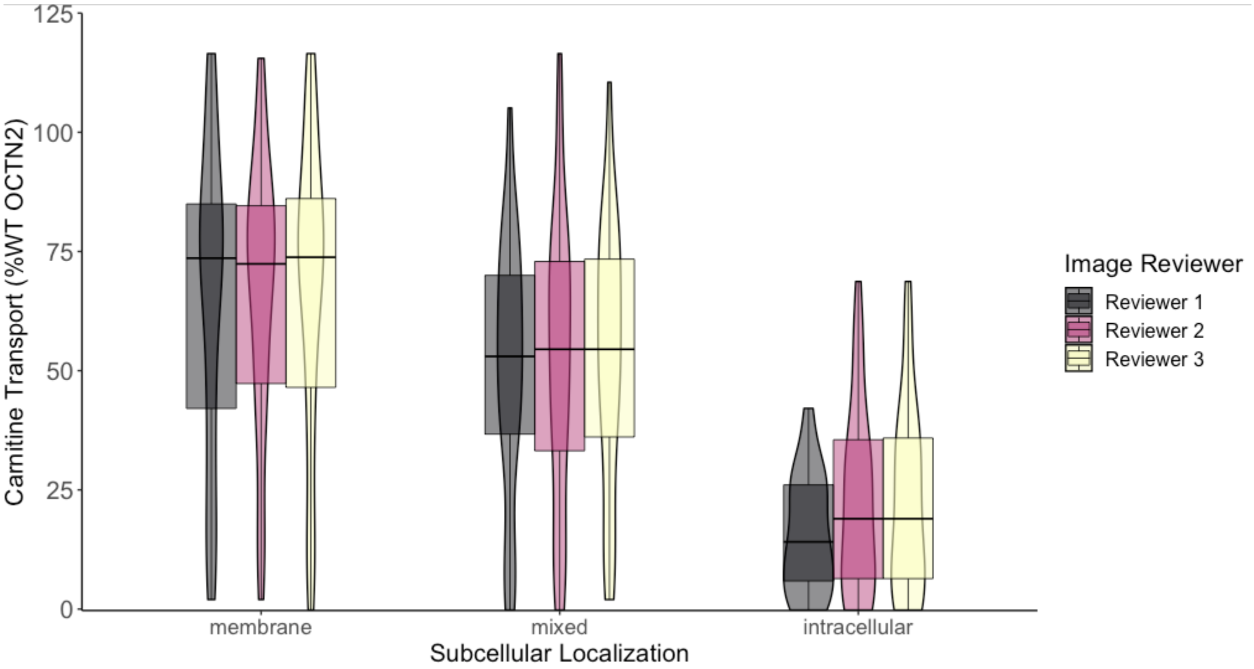

Figure S10. Concordance of subcellular localization of GFP-tagged OCTN2 variants classified by three independent image reviewers blinded to the variant name or function.

Table S1. Constructs from the Mammalian Toolkit used in the generation of SLC22A5 constructs in this study (26).

**MTK part type**	**Description**	**DNA topology**	**Resistance**
MTK1_001	Encodes ConS connector	Circular	Chloramphenicol
MTK2_023	Encodes pCMV-H promoter	Circular	Chloramphenicol
MTK3a_030	Encodes start codon and 6xHIS 3xFLAG	Circular	Chloramphenicol
MTK0_027-SLC22A5 (part 3b)	Encodes SLC22A5	Circular	Chloramphenicol
MTK4a_015	Encodes msfGFP	Circular	Chloramphenicol
MTK4b_001	Encodes BghPA	Circular	Chloramphenicol
MTK5_006	Encodes ConRE connector	Circular	Chloramphenicol
MTK678_001	Encodes ColE1-AmpR backbone vector	Circular	Ampicillin
MTK0_017	Encodes BxB1 attB KanR destination vector	Circular	Kanamycin
MTK0_027	Encodes Part Entry Vector	Circular	Chloramphenicol
JPF0335	Encodes BxB1 recombinase (pCAG_NLS_HA_Bxb1_Addgene51271)	Circular	Ampicillin
SLC22A5 transcriptional unit	SLC22A5 CDS with adaptor sequences for Golden Gate Cloning	Linear	NA

Table S2. Machine learning features.

Feature set	Feature
Sequence-based	Change in polarity
	Change in charge
	Change in mass
	Change in hydrophobicity
	Buried accessible surface area
	Van del Waals forces
	Helix potential
	Residue position
	2D-structural domain (intracellular loop, extracellular loop, transmembrane domain)
Structure-based	Solvent access area
	Solvent access relative score
	Network centrality degree
	Network centrality cluster coefficient
	Network centrality closeness
	Network centrality betweenness
	Network centrality eigenvector centrality
	Network centrality average neighbor degree
	AlphaHelix/turn/coil
Prediction-based	Triad ddG
	AF2 confidence
	ESM-1v
	MSA Transformer
	DeepSequence
	EVE
	Triad DDG
	AF2 confidence
	REVEL
	CADD
	Rhapsody score
	DynaMut2 score
	Potts_EVE
	Potts_hhblits
	Potts_100vertebrates
	Potts_deepsequence
	Potts_30mammals

Dataset S1 (separate file). OCTN2 variants characterized in the study, including function, localization, statistical significance, associated features.

Dataset S2 (separate file). Functional predictions for all 10,583 missense variants in OCTN2. "mean_pred" is a score that represents the probability that the variant is functional (>20% WT OCTN2 carnitine transport). The "deleterious" column binarizes the mean_pred score based on a cutoff that maximizes specificity and sensitivity. A deleterious score of 0 indicates variant is functional; a score of 1 indicates the variant is predicted to be loss-of-function (<20% WT OCTN2 carnitine transport).

Dataset S3 (separate file). Novel OCTN2 variants identified in newborn screening characterized to determine performance of model. Variants were characterized by functional studies with ^{14}C-carnitine and function is expressed as a percent of wild-type OCTN2 carnitine transport.

Dataset S4 (separate file). Clinical variants assayed in this study identified in individuals with confirmed or suspected CTD.

Dataset S5 (separate file). Minimum estimated CTD carrier frequency by ancestral population calculated from population-specific allele frequency of loss-of-function variants. In each population, there were *n* LOF alleles. We estimated the minimum population frequency of a LOF allele (*q)* to be the sum of the population-specific allele frequencies for those *n* alleles. We used the Hardy-Weinberg principle to estimate the minimum carrier frequency of LOF variants included in this study to be $2*(1 - q)*q$.

Dataset S6 (separate file). Global and population-specific allele frequencies of all 150 OCTN2 variants assayed in this study. Data are from gnomAD v2.1.1.

Dataset S7 (separate file). OCTN2 variants with function reported in the literature not assayed in our study. These literature variants were used in evaluation of machine learning models as an additional test/validation set. 82 unique variants had reported function. 12 variants had multiple measurements reported by different publications, for a total of 94 entries. Function was averaged for variants with multiple measurements.

SI References

1. K. J. Karczewski *et al.*, The mutational constraint spectrum quantified from variation in 141,456 humans. *Nature* **581**, 434-443 (2020).
2. I. Adzhubei, D. M. Jordan, S. R. Sunyaev, Predicting functional effect of human missense mutations using PolyPhen-2. *Curr Protoc Hum Genet* **Chapter 7**, Unit7 20 (2013).
3. P. Rentzsch, D. Witten, G. M. Cooper, J. Shendure, M. Kircher, CADD: predicting the deleteriousness of variants throughout the human genome. *Nucleic Acids Res* **47**, D886-D894 (2019).
4. N. M. Ioannidis *et al.*, REVEL: An Ensemble Method for Predicting the Pathogenicity of Rare Missense Variants. *Am J Hum Genet* **99**, 877-885 (2016).
5. S. L *et al.*, Predicting the clinical impact of human mutation with deep neural networks. *Nature genetics* **50**, 1161-1170 (2018).
6. J. Jumper *et al.*, Highly accurate protein structure prediction with AlphaFold. *Nature* **596**, 583-589 (2021).
7. C. H. M. Rodrigues, D. E. V. Pires, D. B. Ascher, DynaMut2: Assessing changes in stability and flexibility upon single and multiple point missense mutations. *Protein Sci* **30**, 60-69 (2021).
8. R. Fraczkiewicz, Exact and efficient analytical calculation of the accessible surface areas and their gradients for macromolecules. *J. Comput. Chem.* **19**, 319-333 (1998).
9. L. Ponzoni, D. A. Penaherrera, Z. N. Oltvai, I. Bahar, Rhapsody: predicting the pathogenicity of human missense variants. *Bioinformatics* **36**, 3084-3092 (2020).
10. C. B, P. N, NAPS: Network Analysis of Protein Structures. *Nucleic acids research* **44**, W375-382 (2016).
11. V. Mariani, M. Biasini, A. Barbato, T. Schwede, lDDT: a local superposition-free score for comparing protein structures and models using distance difference tests. *Bioinformatics* **29**, 2722-2728 (2013).
12. J. Meier *et al.*, Language models enable zero-shot prediction of the effects of mutations on protein function. *bioRxiv* https://doi.org/10.1101/2021.07.09.450648, 2021.2007.2009.450648 (2021).
13. A. J. Riesselman, J. B. Ingraham, D. S. Marks, Deep generative models of genetic variation capture the effects of mutations. *Nat Methods* **15**, 816-822 (2018).
14. T. A. Hopf *et al.*, Mutation effects predicted from sequence co-variation. *Nat Biotechnol* **35**, 128-135 (2017).
15. J. Frazer *et al.*, Disease variant prediction with deep generative models of evolutionary data. *Nature* **599**, 91-95 (2021).
16. R. M. Rao *et al.*, "MSA Transformer" in Proceedings of the 38th International Conference on Machine Learning, M. Meila, T. Zhang, Eds. (PMLR, 2021), vol. 139, chap. 8844-8856.
17. J. Yang *et al.*, Improved protein structure prediction using predicted interresidue orientations. *Proc Natl Acad Sci U S A* **117**, 1496-1503 (2020).
18. S. R. Eddy, Accelerated Profile HMM Searches. *PLoS Comput Biol* **7**, e1002195 (2011).
19. S. Ovchinnikov, H. Kamisetty, D. Baker, Robust and accurate prediction of residue-residue interactions across protein interfaces using evolutionary information. *Elife* **3**, e02030 (2014).
20. D. S. Marks *et al.*, Protein 3D structure computed from evolutionary sequence variation. *PLoS One* **6**, e28766 (2011).
21. M. Figliuzzi, P. Barrat-Charlaix, M. Weigt, How Pairwise Coevolutionary Models Capture the Collective Residue Variability in Proteins? *Mol Biol Evol* **35**, 1821 (2018).
22. C. Hsu, H. Nisonoff, C. Fannjiang, J. Listgarten, Learning protein fitness models from evolutionary and assay-labeled data. *Nat Biotechnol* 10.1038/s41587-021-01146-5 (2022).

23. W. P. Russ *et al.*, An evolution-based model for designing chorismate mutase enzymes. *Science* **369**, 440-445 (2020).
24. H. Kamisetty, S. Ovchinnikov, D. Baker, Assessing the utility of coevolution-based residue-residue contact predictions in a sequence- and structure-rich era. *Proc Natl Acad Sci U S A* **110**, 15674-15679 (2013).
25. D. P. Kingma, J. Ba, Adam: A Method for Stochastic Optimization. *arXiv* (2014).
26. J. P. Fonseca *et al.*, A Toolkit for Rapid Modular Construction of Biological Circuits in Mammalian Cells. *ACS Synth Biol* **8**, 2593-2606 (2019).
